# Expression of concern: Designing a novel visible-light-driven heterostructure Ni–ZnO/S-g-C_3_N_4_ photocatalyst for coloured pollutant degradation

**DOI:** 10.1039/d4ra90105c

**Published:** 2024-09-25

**Authors:** Ali Bahadur, Shahid Iqbal, Hashem O. Alsaab, Nasser S. Awwad, Hala A. Ibrahium

**Affiliations:** a Department of Transdisciplinary Studies, Graduate School of Convergence Science and Technology, Seoul National University Seoul 08826 South Korea; b Department of Chemistry, School of Natural Sciences (SNS), National University of Science and Technology (NUST) H-12 Islamabad 46000 Pakistan shahidiqbal.chem@sns.nust.edu.pk; c Department of Pharmaceutics and Pharmaceutical Technology, Taif University P. O. Box 11099 Taif 21944 Saudi Arabia; d Research Center for Advanced Materials Science (RCAMS), King Khalid University P. O. Box 9004 Abha 61413 Saudi Arabia; e Department of Semi Pilot Plant, Nuclear Materials Authority P. O. Box 530 El Maadi Egypt

## Abstract

Expression of concern for ‘Designing a novel visible-light-driven heterostructure Ni–ZnO/S-g-C_3_N_4_ photocatalyst for coloured pollutant degradation’ by Ali Bahadur *et al.*, *RSC Adv.*, 2021, **11**, 36518–36527, https://doi.org/10.1039/d0ra09390d.

*RSC Advances* is publishing this expression of concern in order to alert readers that concerns have been raised over the integrity of the data published in this article. The authors have been contacted but have not provided the requested raw data. An expression of concern will continue to be associated with the article until a conclusive outcome is reached.

Laura Fisher

17th September 2024

Executive Editor, *RSC Advances*

